# Predicting Condom Use among Undergraduate Students Based on the Theory of Planned Behaviour, Coquimbo, Chile, 2016

**DOI:** 10.3390/ijerph15081689

**Published:** 2018-08-08

**Authors:** Patricio Ramírez-Correa, Muriel Ramírez-Santana

**Affiliations:** 1 Engineering School, Universidad Católica del Norte, Larrondo 1281, Coquimbo, Chile; patricio.ramirez@ucn.cl; 2 Department of Public Health, Faculty of Medicine, Universidad Católica del Norte, Larrondo 1281, Coquimbo, Chile

**Keywords:** condoms, behaviour, Latin America, students

## Abstract

*Background:* Sexually transmitted infections and pregnancy in adolescents are acknowledged public health problems in many countries. Although it is known that the proper use of condoms allows avoiding these health problems, their use in Chile is still limited, for unknown reasons. *Objective*: Based on planned behavioural theory, the aim was to validate a behaviour model regarding condom use by measuring the influence of the variables that predict this use among Chilean university students. *Methods*: A cross-sectional descriptive study was carried out in October 2016 among 151 Chilean university students belonging to the health and engineering areas. The information was collected through a self-administered survey. The sample was divided into two groups: stable and not stable relationships. Partial least squares (PLS) regression was used for the analysis. *Results*: It was possible to explain the condom use of the students by 57%. The attitude was the main variable related to the intention of using condoms, together with the perceived behavioural control. Additionally, there are statistically significant differences in the variables that predict condom use among students with stable relations compared to those without a stable relationship. *Conclusions*: The planned behavioural theory is useful for predicting condom use behaviour when students have a stable partner.

## 1. Introduction

Sexually transmitted infections (STIs) are varied and highly frequent among young people. The World Health Organisation (WHO) estimates that over 357 million STIs occur annually in the world, equivalent to one million infections per day [1]. These infections not only have an acute effect, but many have later repercussions in life, with chronic conditions, including AIDS and cancers, such as those of the cervix and the liver caused by viral infections, for instance the human papilloma virus and hepatitis B and C, respectively. Also, infections such as syphilis have serious long-term consequences (without treatment) and for children’s health, as is the case of congenital syphilis.

In Chile, STIs in young adults and unwanted teenage pregnancy are important public health problems. Chilean epidemiological surveillance systems show rising incidence rates of syphilis, gonorrhoea and HIV in recent years. The incidence is higher in populations between 20 and 34 years old [2,3,4,5]. In fact, the latest study of the burden of disease in the country shows that STIs (excluding HIV/AIDS) are the seventh cause of loss of healthy life years (DALYs) within the group of infectious diseases among men, with HIV/AIDS being the first cause among men, the seventh cause among women and the third cause in the population in general [6]. HIV infections have increased by 67.8% in Chile in the last ten years, with young adults by far leading the numbers. “Due to the lack of prevention campaigns and the difficulties of carrying out the test—an option that the WHO considers key to dealing with the epidemic—there has also been a relaxation in the protection measures for the new generations born in an era when HIV is not a deadly disease” [7]. According to UNAIDS, in the year 2016, Chile had 5000 new HIV infections and there were 61,000 people living with HIV [8]. On the other hand, 5% of new-borns are from mothers between 15 and 19 years old; a situation that creates risky conditions for both the mothers and the new-borns [9].

Condoms are considered a method with a proven effectiveness for preventing pregnancy and the transmission of diseases, and the promotion of their use as a prevention strategy in programmes of good effectiveness is necessary [10]. According to the WHO, when used correctly and consistently, condoms are one of the most effective methods of protection against STIs, including HIV. In fact, their proper use helps to reduce the risk of HIV infection by up to 96%. However, in Chile the low acceptance negatively alters their effect on the reduction of STIs and unwanted pregnancies at the population level. According to the National Health Survey (NHS 2010), only 53.7% of respondents have ever used condoms in their lives, with women using them less than men (50% versus 57%) [11]. In 2016, the NHS showed that only 12% of men and 7% of women reported having used condoms in the past year [12]. Moreover, the same survey showed that 22% of people between 15 to 24 years old had used condoms during the past 12 months [12].

In addition to access to condoms, there are several factors that influence their use being accepted (or not) by young people. Several authors point out that these factors are related to the educational level, gender, information, type of relationship, beliefs, benefits, and the positive/negative effects of use/non-use [13,14,15,16]. In this sense, preventive campaigns concerning unwanted pregnancies and STIs should be directed towards promoting healthy sexual habits in the young population, including the appropriate use of condoms, with messages based on knowledge of the factors that could effectively influence their use. What are the causes of the low levels of condom acceptance in Chile? Which variables determine the behaviour of condom use?

Framed within the study of human behaviour, the theory of planned behaviour (TPB) constitutes a widely accepted conceptual framework for understanding a varied set of behaviours and, specifically, the use of condoms [13]. The TPB was developed by Icek Ajzen in 1985 as an extension of the theory of reasoned action proposed five years earlier by himself and Martin Fishbein [17]. The TPB is also used to design effective interventions and promote behaviour change [13]. According to the TPB, the act of an individual is determined by the intention to perform this behaviour. This intention is a function of the attitude towards the procedure, the subjective norms, and the perceived social control. The attitude represents the individual’s positive or negative feelings about performing a certain action, while the intention describes the strength of the purpose of performing it. Subjective norms represent the individual’s perception of social pressures to perform or not perform a conduct. Finally, perceived behavioural control refers to the individual’s perception of his/her ability to act in a certain way. Considering the antecedents, this research wishes to advance the explanation of the use of condoms at an individual level in Chile. In particular, and based on the TPB, this study aims to measure the influence of the variables that predict the use of condoms in university students in Coquimbo, Chile, 2016.

**Figure 1:**
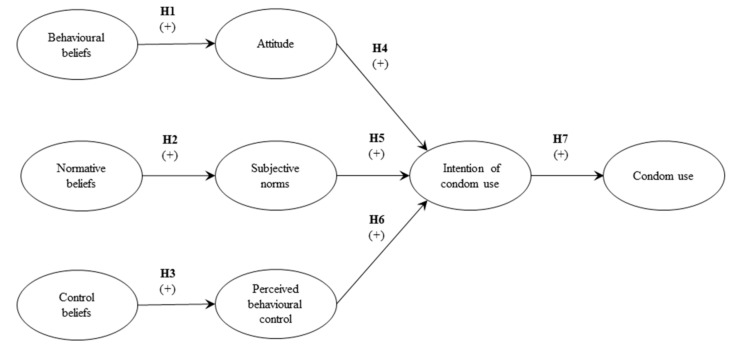
Research model.

For the purposes of the study, and in relation to the individual behaviour of Chilean university students, the research model shown in Figure 1 was constructed. The model, based on the previous literature [14,18], contains the following hypotheses: H1: Behavioural beliefs are positively associated with attitude in relation to condom use. H2: Normative beliefs are positively associated with subjective norms in relation to the use of condoms. H3: Control beliefs are positively associated with perceived behavioural control in relation to condom use. H4: The attitude is positively associated with the intention of condom use. H5: Subjective norms are positively associated with the intention of condom use. H6: Perceived social control is positively associated with the intention to use a condom. H7: The intention of condom use is positively associated with condom use.

## 2. Materials and Methods

A descriptive cross-sectional study was conducted in October 2016. A self-administered survey through the Internet was used to collect the information. The group included 1450 university students, over 18 years old, from the Faculty of Medicine and the School of Engineering of the Guayacán Campus of the Universidad Católica del Norte (Catholic University of the North) (Chile). Convenience sampling was used: individuals corresponding to the needs of the study, namely university students from the health careers and engineering faculties of the institution at which the researchers work, who were willing to participate in and answer the survey. The sample of the study was made up of 151 students. The respondents expressed their explicit consent on the survey website before completing the survey. This survey asked the students their age, sex (two options, male or female), sexual orientation (two options, heterosexual or non-heterosexual), if they had been sexually active in the last month (two options, yes or no), if they had a history of STIs (two options, yes or no), if they had been tested for HIV (two options, yes or no), the use of alcohol or drugs when having sex (three options, never, sometimes or always), and the type of relationship (three options, stable partner for more than three months, open relationship or casual partners, or no partner in the last three months). Additionally, the survey was based on measurement scales validated in the literature [13,18]. The response format was a seven-point Likert-type, where the answers range from “*strongly disagree*” to “*strongly agree*”.

Table 1 shows the items of the measurement scales and the validation carried out for each of the scales, indicating the validity of the measurement scales and the validity values of each item within them. In general, there was a good degree of validity for all the constructs, with average variance extracted values over 0.5 and values of discriminant validity, composite reliability and a Cronbach’s Alpha over 0.7. The values were maintained when the population was divided into two groups according to the type of relationship, a group called “*With stable couple*”, which includes students who had a stable partner for more than three months and a second group called “*No stable couple*”, which integrates students with an open relationship or casual partners. The descriptive analysis was carried out through relative frequencies (sex, sexual orientation, sexually active, condom use, history of STIs, HIV test, type of relationship, use of alcohol or drugs when they have sex). Structural Equations Modelling (SEM) was used as a statistical technique for the analysis of the variables of interest. In particular, the research model and the proposed relationships were analysed with the Partial Least Squares partial square regression technique, (PLS) [19], using the *SmartPLS 3.0* software [17]. PLS is the appropriate option for studies with a small sample, given that it has no restrictions in relation to the normality of the data to be examined. The data collected was analysed in general and the sample was separated, depending on whether the subjects had a stable partner or not. This separation of groups has already been proposed in the literature [20]. The nonparametric Mann–Whitney U test was used in order to prove the existence of median differences between these two groups.

Ethical considerations: All the subjects gave their informed consent before they participated in the study. The study was conducted in accordance with the Declaration of Helsinki, and the protocol was approved by the Ethics Committee of the Faculty of Medicine of the Catholic University of the North (Resolution No. 47 of 28 September 2016), guaranteeing the protection of the ethical principles for research declared by the committee. The field phase was carried out during the month of October, 2016. The application of surveys was configured so that the anonymity of the respondent was preserved at all times. In the introduction of the instrument, the objective was explained and informed consent was included. This had to be read by the student and accepted before answering the questions of the questionnaire. The configuration of the instrument gave the option to withdraw from responding at any time, without sending the questionnaire to the research database.

## 3. Results

The majority of completed surveys were from women (63%) and the average age was 22 years old. Almost 95% had a heterosexual orientation and 80% had been sexually active in the last month (n = 121). The latter was the sample from which the results of the analysis with PLS are presented. Among them, 31% never used a condom in the last month; 21% used one always and about half, sometimes. A low proportion of the respondents reported having a history of episodes of sexually transmitted infections (STIs 6%) and 13% reported having been screened for HIV infection. A relative majority of the respondents (63.5%) stated that they were in a stable relationship (having lasted over three months); 18.5% had not had a partner in the last three months and another 18% mentioned having an open relationship. The consumption of alcohol or drugs when having sex was rare (6%). Of those who reported consumption, 38% mentioned sometimes, and 56% reported never consumed alcohol when having sex. See Table 2 for more detail.

**Figure 2:**
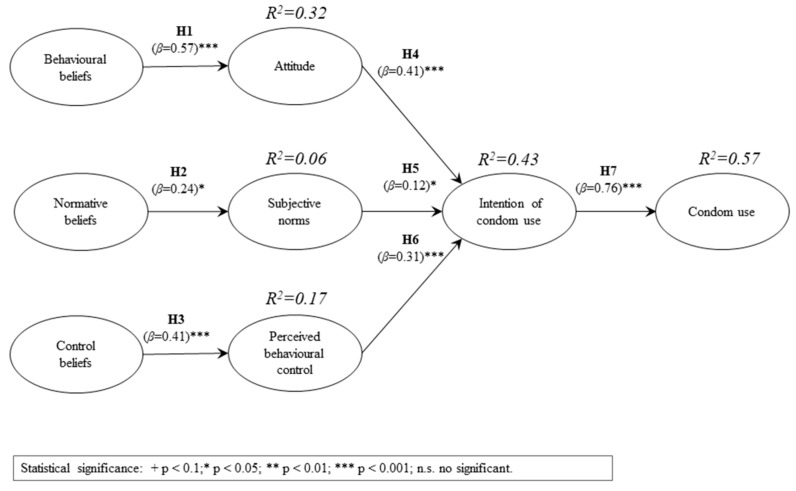
Model for sexually active individuals.

Figure 2 and Table 3 indicate the results with respect to the research model and the relationships proposed. In Figure 2, the R^2^ indicates the amount of the variance of the dependent variables that is explained by the variables that predict it (an R^2^ close to 1 indicates a high predictive power). In Figure 2 and Table 3, the β coefficients (standardised regression weights) indicate the extent to which the independent variables contribute to the explained variance of the dependent variables. The significance of the β coefficients was calculated using Bootstrap (a procedure that creates K sets of samples in order to obtain K estimates of each parameter in the PLS model). The results obtained with Bootstrap were compared with the value of the Student’s t distribution with K-1 degrees of freedom.

In relation to sexually active individuals, the results of the PLS analysis supported the existence of all the relationships proposed in the research model. The strongest relationship found was between the intention to use and the use of a condom. A total of 57% of the use was explained by having the intention to do so. Thus, behavioural beliefs explain 32% of the attitude. Control beliefs explain 17% of the perceived behavioural control and together the attitude, the subjective norms and the perceived behavioural control explain the intention of use in 43%. The relationship between normative beliefs and subjective norms, as well as the connection between the latter and the intention to use, are supported with less statistical significance.

The results changed when the population was separated according to the type of relationship. Firstly, the Mann–Whitney U test indicates significant differences in both the median intention of use of the group without a stable partner, of 6.3, versus the median of the group with a stable partner, of 4.0 (U = 494.5 and *p* = 0.000); and in the variable use of a condom, where the median of the group without a stable partner was 4.0 versus the median in the group with a stable partner of 2.0 (U = 555.0 and *p* = 0.001). Secondly, and in relation to the model, there was a significant difference in the β coefficients associated with the three model relationships: the relationship between normative beliefs and subjective norms, the relationship between attitude and intention to use, and the relationship between the intention to use and use of a condom. In those individuals without a stable partner, only β coefficients associated with two model relationships had a significant value: the relationship between behavioural beliefs and attitude, and the relationship between control beliefs and perceived behavioural control. Meanwhile, in those who had a stable partner the model maintained its significance for all β coefficients.

## 4. Discussion

This study is a contribution to filling the lack of research on this subject in Latin America. In effect, the last meta-analysis of the phenomenon contains only 2% of samples belonging to this geographical area [13]. In relation to the sample, it is highlighted that the percentage of condom users is similar to that reported in previous studies in the third world [21,22,23]. This stationary state contrasts with the increase in social freedoms in recent decades in these nations. In relation to the variables that predict the use of condoms, the study was able to verify that the proposed model is valid for the sample. While all the hypotheses are supported, the variable that has the greatest predictive force is the attitude towards use, followed by the variable of perceived behavioural control. The variable with the lowest incidence in this prediction is the subjective norm. The previous order relation is consistent with prior studies [13,16]. In particular, given that the attitude towards use is the most important variable to explain the intention of use/use of condoms, the association between that variable and the way in which the use of a condom affects pleasure or not should be noted. Particularly, it not only matters whether it is considered good or bad, but also whether or not it is nice to use a condom. In addition, as behavioural beliefs affect that attitude, the beliefs associated with pleasure and the physical and emotional closeness of the couple increase, improving the attitude towards use. Thus, an adequate message to promote the use of condoms among students would be that condoms do not affect pleasure and that it maintains the couple’s closeness and affective bond.

On the other hand, and according to the results, normative behaviours, that is, what friends and parents think, affect the intention of condom use very little. Additionally, religion does not affect anything, and nor does the knowledge that its use prevents STIs or pregnancy. More important than normative conducts is the perceived control over the behaviour; that is, access to condoms. This result coincides with that reported by Bolaños [24] and Reineke et al. (cited by [13]). Therefore, if access to condoms is perceived as close, there is a greater intention to use them. Then, preventive campaigns for young people should aim to improve effective access to condoms, rather than just information. The findings of this study indicate that promoting the timely availability of condoms among young people implies not only improving physical access (dispensing machines in pubs, discos, lyceums, and delivery times in health centres), but also the monetary one (low cost or free); having a condom *“on hand”* at the time of need influences the intention to use.

Another result of interest is the verification of statistically significant differences in relation to the predictors of condom use between the behaviour of students with a stable partner and that of those without a stable partner. These differences are consistent with previous studies [14,15,24]. In particular, the model best predicts condom use behaviour when students have a stable partner; however, in those who do not have a stable partner, neither attitude nor social norms nor social control explains the intention to use condoms. Although the number of students in the sporadic sample is small, given that this group presents a higher risk of infection, the lack of predictors of condom use is worrisome. However, in this group the predictors of social beliefs and control beliefs remain significant. Therefore, considering that marketing strategies are increasingly accepted by the healthcare world [24], preventive messages should emphasise that the use of condoms does not affect pleasure; while improving access to condoms would also serve for this group. Considering the above, and that the use of condoms as a means of prevention is an effective strategy in the reduction of HIV and other STIs, communication strategies must be appropriate to the context and type of the target population [25,26].

Finally, three important limitations of this study are highlighted. First, the sample was taken at a single moment in time. Second, the study population is university students. Third, there are more women than men and the declaration of the sexual behaviour is mostly heterosexual, and probably over-represented. Therefore, its results cannot be extrapolated to the general population. It is expected that these limitations will be overcome in the future with a population-based study.

## 5. Conclusions

The results show that the variables that best predict the use of condoms in this sample of university students were the attitude (associated with the perception of pleasure and feeling close to the partner) and the perceived control (associated with access to condoms). The results suggest that in the case of these Chilean university students, on the one hand, greater accessibility would motivate the use of condoms, and on the other hand, the communication message should emphasise that the use of condoms does not affect the pleasure in sexual intercourse nor does it influence the couple’s affective relationship.

## Figures and Tables

**Table 1:** Cronbach’s alpha, average variance extracted (AVE), composite reliability and weights.

Variables/Items		Sexually Active	With Stable Couple	No Stable Couple
Behavioural beliefs	AVE	0.69	0.64	0.70
	Composite Reliability	0.87	0.84	0.88
	Cronbach’s Alpha	0.78	0.73	0.80
	The use of a condom every time I have sex next month can physically distance me from my sexual partner	0.85	0.84	0.87
	The use of a condom every time I have sex next month can emotionally distance me from my sexual partner	0.86	0.87	0.78
	The use of a condom every time I have sex next month can affect body pleasure (the sensation)	0.78	0.80	0.74
Attitude	AVE	0.69	0.55	0.73
	Composite Reliability	0.87	0.79	0.89
	Cronbach’s Alpha	0.77	0.58	0.81
	For me, the use of a condom every time I have sex in the next month will be…Enjoyable–unenjoyable	0.90	0.92	0.88
	For me, the use of a condom every time I have sex in the next month will be…Pleasant-unpleasant	0.87	0.93	0.65
	For me, the use of a condom every time I have sex in the next month will be…Good-bad	0.70	0.70	0.69
Normative beliefs	AVE	0.74	0.71	0.78
	Composite Reliability	0.85	0.83	0.88
	Cronbach’s Alpha	0.68	0.60	0.72
	My parents (or equivalent to me) think I should use a condom every time I have sex next month	0.94	0.92	0.79
	My closest friends think I should use a condom every time I have sex next month	0.78	0.84	0.89
Subjective norms	People whose opinions I value would approve/disapprove my condom use every time I have sex next month.	N.C.	N.C.	N.C.
Control beliefs	AVE	0.63	0.53	0.63
	Composite Reliability	0.84	0.77	0.84
	Cronbach’s Alpha	0.73	0.55	0.74
	I think that my main sexual partner will oppose us using a condom every time we have sex in the next two months	0.82	0.85	0.57
	Condoms will be easily accessible to me, if I decide to have sex in the next two months	0.79	0.77	0.84
	For me, the use of a condom every time I have sex in the next two months is expensive	0.77	0.76	0.75
Perceived behavioural control	AVE	0.61	0.67	0.60
	Composite Reliability	0.82	0.86	0.82
	Cronbach’s Alpha	0.69	0.76	0.67
	For me, the use of a condom every time I have sex next month is possible	0.85	0.84	0.85
	I am confident that if I wanted to, I could use a condom every time I have sex next month	0.71	0.69	0.84
	It’s easy for me to use a condom every time I have sex next month	0.77	0.78	0.76
Intention of use	AVE	0.96	0.88	0.95
	Composite Reliability	0.99	0.96	0.98
	Cronbach’s Alpha	0.98	0.96	0.98
	I intend to use a condom every time I have sex next month	0.97	0.97	0.99
	I will use a condom every time I have sex next month	0.98	0.98	0.96
	I will try to use a condom every time I have sex next month	0.98	0.98	0.85

Note: N.C. not possible to calculate.

**Table 2:** Distribution of the variables of interest in university students. Coquimbo, Chile, 2016.

Variable		N	%
Sex			
	Masculine	56	37.1
	Feminine	95	62.9
	Total	151	100
Sexual orientation			
	Heterosexual	143	94.7
	No heterosexual	8	5.3
	Total	151	100
Sexually actives (last month)		121	80.1
Condom use (in sexually active)			
	Never	38	31.4
	Sometimes	58	47.9
	Always	25	20.7
	Total	121	80.1
History of STIs (n = 151)		13	8.6
HIV test (at some time)		20	13.2
Type of relationship (n = 151)			
	Stable couple >3 months	96	63.5
	Open relationship, occasional couples	27	17.9
	No couple (last 3 months)	28	18.5
	Total	151	100
Use of alcohol or drugs when having sex			
	Never	85	56.3
	Always	9	6
	Sometimes	57	37.8
	Total	151	100
Age		Mean 21.9 ± 2.96	
		Range 18–34 years	

**Table 3:** Relationship by β coefficients.

Relationship	Sexually Active (Sig.)	With Stable Couple (Sig.)	No Stable Couple (Sig.)	Test of Differences (*p* Value)
Behavioural beliefs ≥ Attitude	0.57 (***)	0.58 (***)	0.55 (***)	n.s.
Normative beliefs ≥ Subjective norms	0.24 (*)	0.33 (***)	−0.13 (n.s.)	*
Control beliefs ≥ Perceived behavioural control	0.41 (***)	0.43 (***)	0.62 (***)	n.s.
Attitude ≥ Intention of use	0.41 (***)	0.60 (***)	0.17 (n.s.)	*
Subjective norms ≥ Intention of use	0.12 (*)	0.12 (*)	−0.13 (n.s.)	+
Perceived behavioural control ≥ Intention of use	0.31 (***)	0.18 (*)	0.00 (n.s.)	n.s.
Intention of use ≥ Condom use	0.76 (***)	0.80 (***)	0.31 (n.s.)	**

Notes: Statistical significance: + *p* < 0.1; * *p* < 0.05; ** *p* < 0.01; *** *p* < 0.001; n.s. no significant.
